# Predictive Models of Life Satisfaction in Older People: A Machine Learning Approach

**DOI:** 10.3390/ijerph20032445

**Published:** 2023-01-30

**Authors:** Xiaofang Shen, Fei Yin, Can Jiao

**Affiliations:** 1 School of Psychology, Shenzhen University, Shenzhen 518060, China; 2 The Shenzhen Humanities & Social Sciences Key Research Bases of the Center for Mental Health, Shenzhen University, Shenzhen 518060, China

**Keywords:** life satisfaction, older adults, machine learning, predictive models

## Abstract

Studies of life satisfaction in older adults have been conducted extensively through empirical research, questionnaires, and theoretical analysis, with the majority of these studies basing their analyses on simple linear relationships between variables. However, most real-life relationships are complex and cannot be approximated with simple correlations. Here, we first investigate predictors correlated with life satisfaction in older adults. Then, machine learning is used to generate several predictive models based on a large sample of older adults (age ≥ 50 years; *n* = 34,630) from the RAND Health and Retirement Study. Results show that subjective social status, positive emotions, and negative emotions are the most critical predictors of life satisfaction. The Support Vector Regression (SVR) model exhibited the highest prediction accuracy for life satisfaction in older individuals among several models, including Multiple Linear Regression (MLR), Ridge Regression (RR), Least Absolute Shrinkage and Selection Operator Regression (LASSO), K Nearest Neighbors (KNN), and Decision Tree Regression (DT) models. Although the KNN and DT models exhibited better model fitting than MLR, RR, and LASSO, their performances were poor in terms of model validation and model generalization. These results indicate that machine learning is superior to simple correlations for understanding life satisfaction among older adults.

## 1. Introduction

Life satisfaction is one of the most important aspects of subjective wellbeing [1] and a key cognitive evaluation component that can be used for its assessment. Life satisfaction is based on a person’s overall evaluation according to their chosen criteria, including whether their social needs have been satisfied, and which results from a judgment about their own life [2,3]. Therefore, life satisfaction becomes a potential factor in psychological adaptation and successful aging in older people [4]. Some studies suggest that life satisfaction is a general measure of attitudes and behaviors toward life at a specific point in time [5,6,7]. Life satisfaction is also considered to be an indicator of positive mental health, and it can help older adults to deal with the difficulties inherent in the life of older people [8]. Life satisfaction in older adults typically declines due to physical and mental disabilities, chronic diseases, or social difficulties that occur in the aging process [9]. Consequently, it is important to explore multiple aspects of life satisfaction in older adults.

Because of chronic diseases and difficulties in the physical mobility, older adults face significant changes in social networks, socioeconomics, health-related issues, and demographic conditions, which all affect life satisfaction [10]. These changes have led to an increase in the research about life satisfaction in older people. To thoroughly understand life satisfaction, most studies have tried to determine the factors that it is affected by, and these typically fall into three categories.

The first category can be described as “individual” factors, including age, gender, and self-reported health status [11,12,13,14]. In particular, these studies conclude that the degree of life satisfaction decreases with age. This is partially explained by living conditions that become more unfavorable due to physical incapacity, low income, reduced social life, and chronic diseases [15]. Physical and psychological health are also associated with life satisfaction. For instance, physical functional impairments and poor cognitive function are more strongly related to low satisfaction [16]. Moreover, studies show that the psychological factors typically reduce life satisfaction in older people include anxiety, depression, loneliness, lack of perceived social support, lack of psychological resilience, personal traits, and other individual psychological factors [17,18,19,20]. Enkvist suggested that the outcome of self-rated health is associated with life satisfaction [21]. Some studies have reported that the level of life satisfaction in older women is lower than that in men because older women are more likely to suffer from chronic gender-related diseases [22,23,24]. Thus, elderly women might rate their health more negatively [25]. However, studies on the relationships between gender and life satisfaction in older people report contradicting results. For example, one study reported no gender difference in life satisfaction [26], Furthermore, Choi and Kim found that monthly income affected life satisfaction in older women only [27], while other researchers reported that income influenced the life satisfaction factor for both genders [28].

The second type of factor that affects life satisfaction in older people is “family”. Specifically, “family” can include marital status, family relationships, financial support from children, family members, family structure, household income, and the other family-related factors [29,30,31,32,33]. Li et al. found that better financial resources and more support from children are associated with higher levels of life satisfaction in older adults [34]. Another study showed that family relationships are an important factor in determining life satisfaction in older adults [35]. Some researchers found that perceived emotional and instrumental support factors from adult children have a negative influence on life satisfaction in older people [36,37]. In contrast, other studies reported that resource support from family was non-significantly related to the level of life satisfaction [38].

The third category that affects life satisfaction in older people can be described as “social”, and includes socioeconomic status, social participation, medical accessibility, and other social elements [13,38,39,40,41]. Kaucic concluded that living conditions, such as housing, the environment, financial position, and safety, as well as physical activity, have a considerable influence on life satisfaction in older people [42]. Several studies found that active engagement, social network, and social support all improve life satisfaction later in life [4,43,44,45]. In contrast, Li and colleagues found that social support was not significantly associated with life satisfaction in older people [46].

Thus, according to the literature, although numerous studies on life satisfaction in older people have been conducted, the findings have been inconsistent in some respects. One reason for this could be related to the methods used to analyze the data; most researchers used regression or mediation methods based on control factors, and the relationship between variables has usually been assumed to be simple linear. However, many real-life factors that affect life satisfaction are complicated; most studies on life satisfaction in the elderly population mainly focus on single or two factors. Moreover, no previous studies have used longitudinal data to predict the risk of life-satisfaction among different elderly people. Thus, it is necessary to pay more attention to the prediction factors from many aspects of life satisfaction on a large sample level.

In fact, many variables cannot be described by simple linear relationships. When this is the case, the results analyzed by using a simple linear fitting are likely unreliable. For example, when a hundred psychological studies published in top journals were repeated, 60% of the conclusions were inconsistent [47,48,49,50]. This phenomenon is an example of the reproducibility crisis facing psychological research at present [51,52]. Redish regarded the reproducibility crisis as a statistical problem, such as “P-hacking” [53]. “P-hacking” refers to nonprincipled decisions obtained during data analysis that aims to reduce the *p*-value of significance tests so that the results appear more reliable than they actually are [54]. It occurs when researchers want to reveal insights that are difficult to observe. P-hacking could help researchers to publish any significant result without a file-drawer problem. The file-drawer problem refers to publication bias where some journals are more likely to accept papers with significant rather than with nonsignificant results. Thus, the negative, neutral, statistically, and non-significant research findings are inaccessible and hidden [54]. However, if model development is achieved through one analysis, false-positive rates with optimized data analysis are ignored, which may lead to the overfitting of the model [55]. As a result, it is difficult to obtain similar results in a high number of repeated studies. Some researchers in the field of psychology focus on explaining the mechanisms underlying behaviors by providing sophisticated theories, but in the process, they often neglect the prediction accuracy of future behavior. Based on this phenomenon, Yarkoni and Westfall suggested that predictive ability is more important than an explanation for understanding human behavior [56]. Consequently, more rigorous scientific research methods, such as machine learning algorithms, have been proposed to increase the reproducibility and predictive accuracy of the research.

Machine Learning (ML) is an evolving branch of computational science that aims to emulate human intelligence by learning from the surrounding environment [57]. Mitchell proposed that ML is a computer program that learns from experience through tasks and performance measures. Compared with traditional data analysis, ML can intelligently perform tasks by learning about the surrounding environment through repeated examples. Machine learning algorithms use input data to produce a particular outcome. These algorithms can automatically adapt their architecture through repetition, similar to experience, to better achieve the desired goal. In the learning process called training, samples of input data are provided together with the expected results. Then, the ML algorithm optimizes its configuration so that it can accurately predict the outcomes from new data that had not been used during training. Therefore, the computer program that can use input data includes features (independent variables) and labels (dependent variables) to provide a fitting model based on the machine learning algorithms. Additionally, the model can accurately predict corresponding labels with features. According to the character of the data, El Napa suggested that machine learning can be categorized into supervised, unsupervised, and semi-supervised. Supervised learning is a prediction method with features (independent variables) and labels (dependent variables). A supervised machine learning algorithm can be used to estimate an unknown dependent variable that is predicted from a given set of known predictors (independent variables). Supervised learning can be divided into two types of algorithms based on the task: regression and classification [57]. An unsupervised machine learning algorithm, such as a clustering analysis, can work on a dependent variable that is predicted by a similar group of data items. Semi-supervised learning, such as that used in image retrieval systems, is a combination of both supervised and unsupervised machine learning. A semi-supervised learning algorithm can use the partially labeled part to infer the unlabeled portion [58]

Although numerous studies have analyzed the relationship between life satisfaction in older adults and various factors, many of them have limited the analyses to simple linear relationships, such as logistic regression models. However, ML can iteratively and simultaneously analyze linear, non-linear, and high-dimensional correlations (data with hundreds or thousands of independent variables) between factors. Therefore, ML can design data-driven models and algorithms with predictive capabilities in an unpredictable approach to achieve a better output. ML not only improves the efficiency and validity of psychological research, but also provides new opportunities for the research conducted in the field of psychology. The principles and techniques of ML can help researchers build predictive models. Because individual, family, and social factors are all closely related to each other, the current study adopts an ML approach to build accurate predictive models of life satisfaction in older adults, based on the identified predictors of life satisfaction. The results should provide a theoretical and empirical basis for increasing life satisfaction in older adults.

## 2. Materials and Methods

### 2.1. Data Source

The data in this study were derived from the RAND (The RAND Center for the Study of Aging) Health and Retirement Study (RAND HRS) wave 10–14 (2010, 2012, 2014, 2016, and 2018) survey. The RAND HRS is a cleaned, easy-to-use, and streamlined data product containing information from the Core and Exit Interviews of the HRS, with derived variables covering a wide range of topics. It contains variables for income and wealth, health insurance policies, work and retirement, economic status, family structure, living conditions, physical performance, life satisfaction, family support, activities of daily living, and mental health. The RAND HRS includes fourteen waves of Core Interview data across sixteen survey years. The baseline survey was conducted in 1992 (wave_1), and follow-up surveys (waves 2–14) were conducted in 1994, 1996, 1998, 2000, 2002, 2004, 2006, 2008, 2010, 2012, 2014, 2016, and 2018 in the United States. The survey participants were people over 50 years old and their spouses. We selected samples for this study from waves 10–14 because the variable structure of the questionnaires for these waves was consistent. The RAND HRS datasets in 2010–2018 were merged according to the sequential arrangement of IDyear, which was named by the database and selected variables. Eventually, 34,630 older adults who responded to the survey were included.

### 2.2. Measurements

#### 2.2.1. Outcome Variable

The level of life satisfaction was assessed by using five questions from the Satisfaction With Life Scale (SWLS) [3]. Response options ranged from 1 (strongly disagree) to 7 (strongly agree), and the answer to each question was considered as a representation of life satisfaction. The Cronbach’s coefficient alpha for this scale ranged between 0.87 and 0.89 [3,59,60,61]. The sample question was: “In most ways my life is close to my ideal”.

#### 2.2.2. Predictors

The predictors used in this study were primarily obtained from the following two categories: (1) Demographic variables, including gender, age, race, economic status, religious preference, marital status, and number of children. The years of education were assessed using one question: How many years of education did you have? The number of children was determined by one question: How many children do you have? (2) Self-assessments included personality type; mood, including emotional disposition (positive/negative), life outlook (optimistic/pessimistic), and hopelessness; subjective social status; degree of social inclusion; social activity; and living conditions.

The big-five personality traits of neuroticism, extroversion, openness, agreeableness, and dutifulness were assessed with the 31 items from both the Midlife in the United States Survey (MIDUS) [62] and the International Personality Item Pool (IPIP) [63]. The sample item was: “I am the life of the party”. The response options ranged from 1 (often) to 7 (hardly ever or never). The Cronbach’s coefficient alpha of the IPIP was 0.66 [62] and the Cronbach’s coefficient alpha of MIDUS was 0.81 [63].

Emotional disposition was measured by the Positive Affect and Negative Affect Schedule (PANAS) [64] that had 25 items that asked how often participants felt positive/negative. The response options ranged from 1 (often) to 7 (hardly ever or never), and the reliabilities of this measurement were 0.89 on the positive scale and 0.85 on the negative scale [65].

The Revised Life Orientation Test [66] has 6 items that measure life outlook as optimistic or pessimistic. The sample item was: “I hardly ever expect things to go my way”. The response options ranged from 0 (strongly disagree) to 4 (strongly agree). The Cronbach’s alpha of this scale was 0.78 [66].

Degree of hopelessness was assessed by a four-item scale. The scale comprised two items from Everson’s hopelessness scale (Cronbach’s coefficient alpha of 0.84) and two items from Beck’s hopelessness scale (Cronbach’s coefficient alpha of 0.88) [67]. The sample item was: “I feel it is impossible for me to reach the goals that I would like to strive for”. The response options ranged from 1 (strongly disagree) to 6 (strongly agree).

Subjective social status was measured using the MacArthur Subjective Social Status Scale. Participants were shown a picture of a 10-rung ladder and asked: “Which rung do you feel represents you?” The scores ranged from 1 (lowest subjective social status) to 10 (highest subjective social status). The Cronbach’s alpha of this scale was 0.81 [68].

Social inclusion was assessed by a seven-item scale that queried social support, the frequency of network contacts, and the number of intimate relationships with spouses, children, relatives, and friends. The Cronbach’s coefficient alpha of this scale ranged between 0.75 and 0.87 [69,70].

Social activity was assessed by twenty questions that were created by investigators from HRS; these items measured the frequency of social activity participation. A sample measure for the average number of social contacts per week was created using responses from the survey question about how often respondents get together with any neighbors or members of their institutional setting just to chat or for a social visit and the unit of time. The response options ranged from 1 (every day) to 7 (never).

Living conditions were measured by the environmental identity scale (Cronbach’s alpha of 0.64) [71] and the neighborhood Cohesion scale (Cronbach’s alpha of 0.86) [72,73], and included 8 items.

### 2.3. Statistical Analysis

Firstly, we used correlation analyses, including the Pearson zero-order correlation and Pearson correlations, to select predictors correlated with life satisfaction. Correlation analyses were conducted with the Statistical Package for Social Science (SPSS, version 24.0; IBM Corp. Armonk, NY, USA). In the point two-column correlation, we coded the dichotomous variables with values of 0 and 1, and then computed a standard Pearson’s correlation between the categorical and continuous variables. Secondly, according to the results obtained from the correlation analyses, we collected and prepared data in a format that could be given as inputs to the machine learning algorithm. Thirdly, we removed some features (independent variables) in the LASSO regression model and obtained the most important predictors through feature selection. Fourthly, we selected the best machine learning algorithms and appropriate values for obtaining the best model fitting results in the training data. Fifthly, we computed the metrics, including Mean Absolute Error (*MAE*), Mean Squared Error (*MSE*), and Coefficient of Determination (*R*^2^) to evaluate the models’ estimation performance in the test data. Lastly, we compared the metrics from different models in the machine learning algorithms. Feature selection, model fitting, model evaluation, and model comparison were conducted in Python using the Scikit-learn project as the machine learning library.

#### 2.3.1. Data Pre-Processing

Data pre-processing is essential before the procedure of model fitting. After pre-processing the missing values and outliers, the data entering the model are cleaner. Before data processing, we selected some core predictors in the RAND HRS data in 2010–2018 and deleted the unrelated variables. These remaining variables were renamed. The data pre-processing conducted in the current study comprised the following steps: (1) In order to make nominal predictors meaningful and better explained in the linear model, we recoded predictors, such as religious preference and marital status, using the one-hot encoding technique, which can code the dummy variables to 1 and 0. (2) The data from waves 10–14 were used in both the training set for model development and the test set for model validation. Specifically, we used the predictors in waves 10–14 to predict the level of life satisfaction in older people. After a correlation analysis, the data that were obtained from predictors unrelated and weakly related (*r* < 0.2) to life satisfaction were removed. Then, 21,928 samples remained for analysis by machine learning algorithms. The data were split into training (*n* = 15,350) and test (*n* = 6578) (a ratio of 7:3) sets.

#### 2.3.2. Processing of Missing Values

Some data related to education, family income, social support, network contact, and intimacy were missing. Thus, we used intermediate values from nearby points to fill in the missing data.

#### 2.3.3. Model Selection

Machine learning can provide the optimal model for classification and prevent overfitting. To compare the ability of different machine learning models to predict the degree of life satisfaction, we first considered supervised learning algorithms, including Multiple Linear Regression (MLR), Ridge Regression (RR), Least Absolute Shrinkage and Selection Operator Regression (LASSO), Support Vector Regression (SVR), K Nearest Neighbors (KNN), and Decision Tree Regression (DT) models. These six regression models are described in detail elsewhere [74].

MLR is a mathematical model that can estimate dependent outputs with multiple variable inputs [75], and uses the least squares method to calculate the coefficients. RR is similar to MLR, but the regression coefficients are derived by shrinkage methods. RR performs L_2_ regularization (i.e., L_2_-penalty) by imposing a penalty that is determined by the sum of squared coefficients. LASSO is a shrinkage method that calculates the coefficient values by imposing a penalty. SVR is the common version of support vector machines and has proper generalization and accuracy. SVR performs finds a good solution by preserving all the main features that characterize the algorithm. KNN is a nonparametric model that uses the k nearest neighbors from a given point. The k parameter can be selected by users. DT can be built on both regression and classification models, and is composed of decision and leaf nodes according to the features and targets. The similarity of DT algorithms to human thinking ability and the tree-like logic flow makes these algorithm structures easy to understand.

#### 2.3.4. Model Evaluation

Different error metrics were used to measure the relationships between the predicted and actual values when evaluating each model’s estimation performance. The general error metrics for regression model evaluation included Mean Absolute Error (*MAE*), Mean Squared Error (*MSE*), Root Mean Square Error (*RMSE*), and Coefficient of Determination (*R*^2^) [76].

*MAE*, *MSE*, and *RMSE* can take values from 0 to infinity, with values closer to 0 indicating more successful predictions. Additionally, *R*^2^ ranges from 0 to 1. *RMSE* is more advantageous for larger datasets. Additionally, the success rate of the estimation model is higher when the accuracy ratio of the *R*^2^ measurement is approximated to 1. Among these error metrics, model estimation accuracy was evaluated using the *R*^2^ value. Algorithm error was evaluated via *MAE* and *RMSE* values.

Equation (1) shows that the Mean Absolute Error (*MAE*) corresponds to the absolute error between actual and predicted values, which is expressed as

(1) $$MAE=\frac{1}{n}\sum_{i=1}^{n}|y_{i}-\hat{y_{i}}|,\;\in \left[0,+\infty \right)$$

where $y_{i}$ is the actual value,$\hat{\;y_{i}}$ is the predicted value, and *n* represents the size of the dataset.

Equations (2) and (3) show that the distance between the predicted values and their actual values on the regression curve is expressed by the residuals, and the standard deviation of the residuals provides the mean square error (*MSE*) and root mean square error (*RMSE*) values. *MSE* and *RMSE* can be calculated as

(2) $$MSE=\frac{1}{n}\sum_{i=1}^{n}{\left(y_{i}-\hat{y_{i}}\right)}^{2},\;\in \left[0,+\infty \right)$$

(3) $$RMSE=\sqrt{\frac{1}{n}\sum_{i=1}^{n}{\left(y_{i}-\hat{y_{i}}\right)}^{2}},\in \left[0,+\infty \right)$$

where $y_{i}$ is the actual value, $\hat{y_{i}}$ is the predictive value, and *n* represents the size of the dataset.

As shown in Equation (4), the expression of the determination coefficient (*R*^2^) is given as

(4) $$R^{2\;}=1-\frac{\sum_{i=1}^{n}{\left(y_{i}-\hat{y_{i}}\right)}^{2}}{\sum_{i=1}^{n}{\left(y_{i}-\bar{y}\right)}^{2}}\in \left[0,1\right]$$

where $y_{i}$ is the actual value, $\hat{y_{i}}$ is the predictive value, *n* represents the size of the dataset, and $\bar{y}$ is mean of *y* values.

The *R*^2^ value indicates how well the model fits by using the ratio of the sum of squared differences between the true and predicted values to the variance of the actual values. *R*^2^ ranges from 0 to 1, with lower values indicating poorer model fits. When *R*^2^ is closer to 1, it indicates that the independent variables explain more of the variance in the model’s dependent variables and is thus a better model fit. Empirically, we can determine that a model fit is good when *R*^2^ is greater than 0.4 and the *p*-value is less than 0.05.

*MAE*, *MSE*, and *R*^2^ were used to evaluate and compare the model fitting effect in the current study.

## 3. Results

### 3.1. Descriptive Statistics with Predictors and Life Satisfaction

Descriptive statistics of predictors are shown in Table 1 and Table 2. Table 1 displays the demographic predictors (individual and family factors), each dimension within a predictor, and the proportion of older people in the RAND HRS (2010–2018) that was associated with each dimension. Table 2 presents the descriptive statistics of the important predictors. The level of life satisfaction in older adults was 24.99 ± 7.56 (*M ± SD*).

### 3.2. Correlations between Predictors and Life Satisfaction

Pearson’s zero-order correlations were conducted between each dimension of the predictors (gender, race, religious preference, and marital status) and life satisfaction. Pearson’s correlations were used to explore the relationship between the other predictors (age, education, household income, number of children, subjective social status, social activities, personality characteristics, social integration, emotional status, and living conditions) and life satisfaction. As shown in Table 3, the analysis results indicate that among the individual demographic predictors, gender, number of children, and religion (Judaism) are not significantly correlated with life satisfaction. Instead, personality traits (agreeableness); positive support from relatives and friends; negative support from relatives and friends; intimacy with children, relatives, and friends; and network contact with children, relatives, and friends are correlated with life satisfaction (*r* < 0.2). Subjective social status, social activities, personality characteristics (dutifulness, openness, extraversion, and neuroticism), positive support from spouse and children, negative support from spouse and children, intimacy with spouse, emotional status, and living conditions all significantly correlated with life satisfaction (*r* > 0.2).

### 3.3. Feature Selection of Predictive Variables

Following the correlation analysis, we selected predictors whose correlation coefficients (*r*) were higher than 0.2. Altman suggested that the relationship between independent and dependent variables is poor when Pearson’s correlation coefficient is less than 0.2. Therefore, we selected the predictors whose correlation coefficients were higher than 0.2. Feature selection that refers to independent variables is the most important part of the process. By selecting important features and removing irrelevant features, feature selection can solve the dimensionality reduction problem, meaning that the number of input variables in a dataset can be reduced, and the predictive models are more concise. LASSO regression is often used for feature selection, which reduces the coefficients of some non-significant predictors to zero by adding a regularization penalty term in the linear regression model. Therefore, we used LASSO regression for feature (independent variables) selection.

In the LASSO model fitting, the regularization term is adjusted for each penalty coefficient *α*. The *α* value is a nonnegative regular parameter and controls the complexity of the model. The larger the *α*, the stronger the penalty, which means that more concise models and fewer features (independent variables) are incorporated. The training- and testing-set indices (*R*^2^ values) of the model fit were evaluated for different values of *α*. The predictors that correlated with life satisfaction were selected for the features, and the results are shown in Table 4.

**Figure 1:**
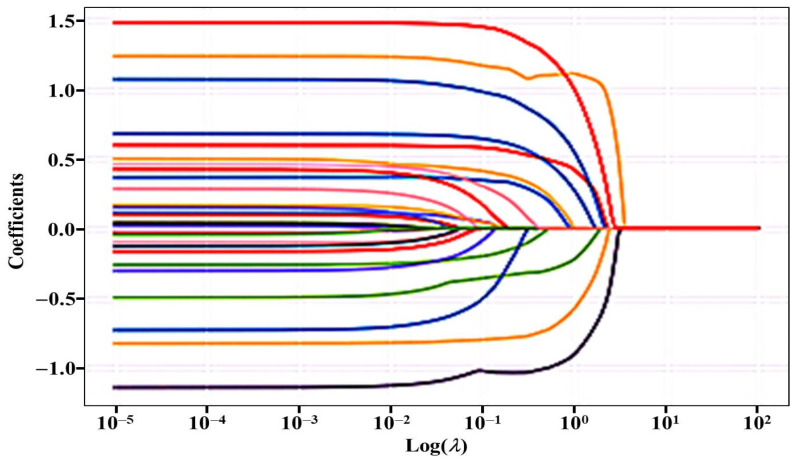
The feature selection of life satisfaction in LASSO regression.

Figure 1 shows that the coefficients for the predictors for both training and test sets tended to stabilize when log (λ) was less than 0.01; the different line colors represent the coefficients from 18 predictors of the value of log (λ). Additionally, the fitting results in Table 4 show that the *R*^2^ value for the model was higher for the test set than for the training set, and that the model performed better on the training set when the *α* values were 0.01 and 0.001. Therefore, the models with *α* values of 0.01 and 0.001 were selected as the best models. By analyzing the predictor coefficients, we observed that none of the 18 predictors were excluded from the LASSO model.

### 3.4. Model Performance

The 21,928 samples were randomly divided into training and test sets in a 7:3 ratio. Thus, the training set comprised 15,350 samples and the test set had 6,578 samples. The 18 selected predictors were included in the model along with life satisfaction. Model fitting (training) and validation (test) results for the three linear regressions models (MLR, RR, and LASSO) were as follows: (1) MLR: *MAE*, *MSE*, and *R*^2^ were 4.392, 32.796, and 0.430, respectively, for the training set and 4.339, 32.579, and 0.433, respectively, for the test set. The correlation between the predicted and true values reached 0.659 for the test. The evaluation of the metrics revealed that the MLR model performance was good for the test set. The model was able to account for 43.33% of the variation in life satisfaction. (2) RR: The RR model’s parameter was *α* in the training set. By parameterizing *α*, we found that this model was optimal when *α* was 0.1. *MAE*, *MSE*, and *R*^2^ were 4.392, 32.796, and 0.430, respectively, for the training set and 4.339, 32.579, and 0.433, respectively, for the test set. The correlation between the predicted and true values reached 0.659 for the test set. The RR model explained 43.33% of the variation in life satisfaction. (3) LASSO regression: LASSO regression also treats *α* as a parameter in the training set. By adjusting *α*, the LASSO model was optimal when *α* was 0.003. *MAE*, *MSE*, and *R*^2^ were 4.392, 32.796, and 0.430, respectively, for the training set and 4.339, 32.579, and 0.433, respectively, for the test set. The correlation between the predicted and true values reached 0.659 for the test set. The LASSO model accounted for 43.33% of the variation in life satisfaction.

The following values are the fitting and validation results for the three nonlinear models (SVR, KNN, and DT). (1) SVR: the parameters were the *C* (penalty parameter) and *γ* (Gaussian kernel) values. When the *C* value is large, the parameter is accurate, and the model has good generalization. The SVR model was optimal when *C* was 10, *γ* was 0.005, and the kernel function was a radial basis function (rbf) that computes that the dot product in the feature (independent variables) significantly affects the performance of the classifiers. SVR can easily deal with nonlinear problems in classification and regression models by using the kernel function. *MAE*, *MSE*, and *R*^2^ were 4.155, 32.173, and 0.441, respectively, for the training set and 4.151, 32.416, and 0.436, respectively, for the test set. The correlation between the predicted and true values was 0.669 for the test set. SVR outperformed the linear models in both model fitting and validation, and explained 43.62% of the variation in life satisfaction. (2) KNN: the KNN parameter is the *K*-value that indicates the number of selected proximity points. The KNN model was optimal when *K* was 9. *MAE*, *MSE*, and *R*^2^ were 4.054, 28.452, and 0.506, respectively, for the training set and 4.488, 34.961, and 0.392, respectively, for the test set. The correlation between the predicted and true values was 0.628 for the test set. The KNN model performed badly on the test set and explained only 39.19% of the variation in life satisfaction. (3) DT: the DT parameters were max_depth, min_samples_leaf, and min_samples_split values. The max_depth is an indication of the depth of the tree. The min_samples_leaf is the minimum number of samples in the node before branching. The min_samples_split refers to the minimum number of samples that are allowed to be branched under the node. The DT model was optimal when the max_depth was 7, the min_samples_leaf was 4, and the min_samples_split was 2. *MAE*, *MSE*, and *R*^2^ were 4.324, 31.815, and 0.447, respectively, for the training set and 4.595, 36.444, and 0.366, respectively, for the test set. The correlation between the predicted and true values was 0.608 for the test set. The DT model performed poorly and explained only 36.61% of the variation in life satisfaction.

### 3.5. Model Evaluation and Comparison

**Figure 2:**
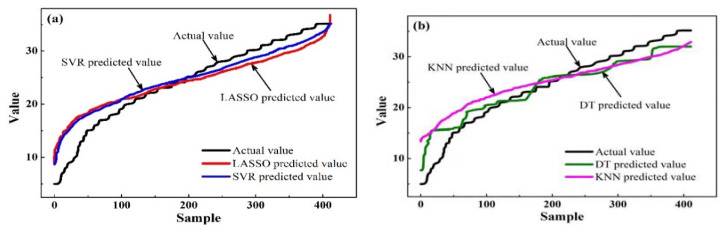
Predictive effect for life satisfaction model validation: (**a**) good performance, (**b**) bad performance. The *x*-axis represents the sample number that was manually coded based on the *y* value, and the *y*-axis represents the *y* values, including actual and predicted values (see Equations (1)–(4)). The curves were drawn after sorting the *y* values.

As shown in Table 5, the comparison of the six models revealed that the three linear models performed more consistently than the nonlinear models. Comparing the fits for the three nonlinear models, SVR, KNN, and DT performed well on the training set, with *R*^2^ reaching 0.441, 0.506, and 0.447, respectively. However, the fit indices for the KNN and DT models were poor for the test set with *R*^2^ values of 0.392 and 0.366, respectively. This indicates that the KNN and DT models exhibited overfitting and poor model generalization abilities. *R*^2^ from the SVR achieved 0.436 on the test set, and performed better on the training set than the MLR, RR and LASSO models. Moreover, the *R*^2^ values of the SVR and LASSO models in the training set were close to those in the test set, respectively. Additionally, the *R*^2^ values of SVR on the training and test sets were both greater than LASSO. Due to the fact that the fit indices of the linear models (MLR, RR, and LASSO) were basically the same in the training and test sets, the prediction performances of the SVR, LASSO, KNN, and DT models were compared, as shown in Figure 2. It can be observed that the predicted values obtained from the SVR and LASSO models were close to the actual values of the samples, and they showed a smoother and more stable trend than those from the KNN and DT models.

In summary, model fitting was good for the MLR, RR, and LASSO models, and excellent for the SVR, KNN and DT models in the training set. Among the six models, the SVR method performed best in model fitting and generalization. Moreover, both the SVR and LASSO models performed well for generalization, and showed better predictions than the KNN and DT models. Therefore, in the terms of predicting life satisfaction, the SVR model is the best predictive model in this study.

### 3.6. Important Feature of Predictors in Different Models

The regression analysis results obtained from the MLR, RR, and LASSO models are identical. In order to explore the key predictors affecting the level of life satisfaction in the elderly, the regression coefficients of the predictors for linear regression were examined. Table 6 shows that the absolute values of the regression coefficients for subjective social status, optimism, positive emotion, and negative emotion when predicting life satisfaction were high.

Because both KNN and SVR are black box models in which the system is entirely based on the data, the relationships between predictors and life satisfaction could not be known. When DT fits the model, the algorithm calculates entropy to discriminate the importance of predictor features. Although model validation was poor in the DT model, that did not affect its ability to estimate the important features of the independent variable predictors. Therefore, with DT regression, the important features can represent relationships between predictors and dependent variables. Table 7 shows that subjective social status, positive emotions, and negative emotions are the most important predictors of life satisfaction.

## 4. Discussion

A correlation analysis followed by LASSO regression indicated 18 predictors associated with life satisfaction in older people, and these were included in six machine learning models as different approaches to studying the predictors of life satisfaction in older adults. We found that the SVR model had the best model fitting among the six models, and also predicted life satisfaction in older adults much better than the three linear models (MLR, RR, and LASSO). Among the nonlinear models, SVR exhibited good validation and generalization abilities, while the KNN and DT models showed poor generalization and overfitting. Additionally, the results of the important features in LASSO and linear regression analyses show that subjective social status, positive emotions, and negative emotions are the most critical predictors of life satisfaction.

The reasons for these results are as follows. First, SVR possesses good properties that differ from the other models. By using the kernel trick that can transfer the training data from the input space to a high-dimensional-, even infinite-dimensional-feature, space via implicitly defined nonlinear mapping, SVR can solve nonlinear problems in arbitrarily high-dimensional-feature spaces that are indexed by multidimensional data structures [77]. We included some classification predictors, such as subjective social status, gender, religion, and race, in the current study. SVR produces lower errors in model fitting, and its algorithm is not affected by extreme values. Therefore, SVR exhibited a good prediction ability and outperformed the linear models (MLR, RR, and LASSO) in terms of model validation. Thus, SVR is stable and good for accurate predictions. Second, the KNN and DT models fit the training dataset well, but performed poorly on the test set. This could be due to overfitting, the sensitivity of these two models to the data, and their instability. Kataria and Singh suggested that KNN is inefficient for large, high-dimensional data [78]. KNN prediction relies on nearby samples and calculates the distance between predictors and life satisfaction. DT performs model fitting by minimizing the entropy and calculating the differences in important features. Moreover, DT is prone to overfitting due to the complex algorithmic rules. DT and KNN models could not predict life satisfaction in older people very well. Third, machine learning algorithms combine attributes in a sophisticated way and are better than simple modeling techniques at meeting the given criteria. Due to the different variable types, there are inconsistent results for the relationship between gender and support provided by family with life satisfaction in older adults. Researchers have suggested that if a significant unstable nonlinearity exists between the predictors and outcome, then machine learning models are probably more suitable than linear models as classifiers [79].

Lastly, the analysis of the important features revealed by the MLR, KNN, and LASSO regression models and the DT model showed that subjective social status, positive emotions, and negative emotions are the most critical predictors of life satisfaction. Life satisfaction is an individual’s overall cognitive evaluation of their life and is an important indicator of active aging in older adults. Subjective social status is an individual’s perceived position or social rank in a group or society and is a crucial predictor of individual life satisfaction [80,81]. Additionally, individuals with a higher social rank are more respected and subsequently have greater life satisfaction. Older people whose social rank is lower might have fewer psychological resources, and exhibit lower self-esteem, lower perceived control, and fewer positive emotional states to deal with problems. Thus, especially for older people with a low subjective social status, insufficient resources make them feel more pressure in life and lead to increased negative emotions. If they can receive more social support (i.e, more resources) or perceive themselves as having a higher social status, it will help them feel positive emotions and greater life satisfaction. Positive emotion directly reflects an individual’s good mental state; thus, it can be an important predictor. Emotions are directly related to life satisfaction and reflect individual attitudes in dealing with daily life [82,83]. The linear model analyses showed that optimism is an important predictor of life satisfaction. However, the importance of optimism for life satisfaction was reduced in the DT model. Optimism is an individual’s positive attitude towards life [84]. Individuals who are optimistic might be more positive when dealing with problems and more sensitive towards positive information. Although, Kapikiran found that the influence of optimism on life satisfaction did not reach significant levels [85], other research has shown that the interaction between optimism and perceived stress affects life satisfaction [86]. Thus, optimism might interact with other predictors to produce a particular effect on life satisfaction. As a consequence, we must focus further on subjective social status and moods in older adults. For example, if older people can perceive more social support despite insufficient resources and poor health, it might help them become happier and have greater life satisfaction.

Overall, the SVR model outperformed the linear models in both the training and test sets, and showed good model fit and generalization abilities. The SVR model could not specify the relationship between the predictors in the model, while the MLR, RR, and LASSO models were used to understand the explicit linear relationship between predictors and life satisfaction. Thus, the exploration of the data by different models should be fully considered, and the model should be comprehensively analyzed by various algorithms as much as possible to improve its accuracy. In the future, studies should test whether combining the MLR, RR, LASSO, and SVR models provides more accurate model fitting results and better predictions. Additionally, we should pay more attention to improve the level of subjective social status and positive emotions among older adults in real life. According to the role of these predictors, we should provide some community activities to improve life satisfaction and active aging.

The present study had some limitations. First, the *R*^2^ values obtained from the six models were between 0.430 and 0.596 in the training set, indicating that the models could explain 43.00% to 59.60% of the variation in life satisfaction, which is a medium-level explanation. Higher percentages might not have been possible to obtain because life satisfaction is influenced by numerous social factors. Although the data were pre-processed, there are still many missing values and data noise, which might have affected the results. In addition, a considerable amount of unrelated information obtained from complex and noisy variables in the RAND HRS could affect model fitting and validation parameters, including the data derived from the database. Thus, each variable obtained from the database is important and we cannot avoid the problem of multicollinearity. Despite these limitations, the results obtained in this study could provide an important contribution to the exploration of life satisfaction in older people.

## 5. Conclusions

In conclusion, MLR, RR, LASSO, and SVR models were proposed to explain the association between numerous predictors and life satisfaction. We observed that subjective social status, positive emotions, and negative emotions were important predictors for life satisfaction. The non-linear SVR model performed better than the three linear models (MLR, RR, and LASSO). The non-linear KNN and DT models exhibited the best model fitting ability but poorest model validation and model generalization factors. In the future, we can use a combination of SVR and linear models with a machine learning algorithm to establish good model fitting and validation for a prediction model of life satisfaction. With the machine learning approaches, we can comprehensively focus on the type, distribution, and relationships between different predictors and life satisfaction. This technique should improve the reproducibility and application of research results regarding life satisfaction among older adults. Additionally, ML can increase the speed of processing.

## Figures and Tables

**Table 1:** The proportion of older adults for demographic predictors (*N* = 34,630).

Predictors	Dimension	Percent of Sample (%)	Predictors	Dimension	Percent of Sample (%)
Gender	Male	42.5	Religion	No religion	19.7
	Female	57.5		Christianity	78.7
Age	AHEAD (<1924)	1.5		Jewish	1.5
	CODA (1924–1930)	5.6		Other religion	0.1
	HRS (1931–1941)	25.7	Number of children	No child	18.5
	WB (1942–1947)	14.6		1–3 children	69.1
	EBB (1948–1953)	20.4		4–6 children	11.0
	MBB (1954–1959)	20.8		≥7 children	1.4
	LBB (1960–1965)	11.4	Family income	Difficulty	26.5
Education level	Elementary education	10.8		Medium	63.0
	Junior high school	20.4		Wealthy	10.5
	Vocational education	29.2	Marital status	Married	59.6
	University graduates	20.2		Separated	2.1
	Masters or above	14.2		Divorced	14.3
Race	Black	23.1		Widowed	17.9
	Other races	76.9		Single	5.9

Notes: AHEAD is the Study of Assets and Health Dynamics cohort (born before 1924), CODA is Children of Depression cohort (1924–1930), HRS is the initial 1992 Health and Retirement Study cohort (born 1931 to 1941), WB is War Baby (born 1942 to 1947), EBB is Early Baby Boomer cohort (born 1948 to 1953), MBB is Mid-Bay Boomer cohort (born 1954 to 1959), and LBB is Late-Baby Boomer cohort (born 1960 to 1965).

**Table 2:** Descriptive statistics for important predictors (*N* = 34,630).

Predictors	Dimension	*M* ± *SD*	Predictors	Dimension	*M* ± *SD*
Positive support	Spouse	10.54 ± 1.85	Personality trait	Dutifulness	32.85 ± 3.97
	Children	9.65 ± 2.22		Openness	20.68 ± 3.90
	Relatives	8.60 ± 2.61		Extroversion	15.89 ± 2.85
	Friends	9.21 ± 2.21		Agreeableness	9.88 ± 5.42
Negative support	Spouse	7.70 ± 2.67		Neuroticism	7.87 ± 2.40
	Children	6.94 ± 2.65	Mood	Optimism	13.64 ± 3.32
	Relatives	6.34 ± 2.53		Pessimistic	7.24 ± 3.67
	Friends	5.65 ± 1.93		Positive emotion	20.82 ± 7.32
Intimacy	Spouse	3.50 ± 0.70		Negative emotion	47.05 ± 10.28
	Children	2.42 ± 2.61		Hopelessness	8.75 ± 4.86
	Relatives	3.48 ± 4.70	Living conditions	Neighborhood relations	21.80 ± 5.24
	Friends	4.26 ± 5.21		Environmental identity	9.88 ± 5.42
Network contact	Children	12.11 ± 3.28	Social activities		92.08 ± 14.79
	Relatives	10.24 ± 3.34	Subjective social status		6.45 ± 1.76
	Friends	11.60 ± 3.22			

*M*, mean scores; *SD*, standard deviation.

**Table 3:** Correlations between life satisfaction and predictors (*r*).

Predictors	Dimension	Life Satisfaction	Predictors	Dimension	Life Satisfaction
Gender		0.007	Positive support	Spouse	0.347 **
Age		−0.098 **		Children	0.243 **
Education		0.123 **		Relatives	0.149 **
Family income		0.095 **		Friends	0.114 **
Number of children		−0.029	Negative support	Spouse	−0.321 **
Race		0.134 **		Children	−0.224 **
Religions	Christianity	−0.061 **		Relatives	−0.190 **
	Jewish	−0.004		Friends	−0.148 **
	No religious	−0.036 **	Intimacy	Spouse	0.320 **
	Other religions	−0.022 **		Children	0.058 **
				Relatives	0.059 **
Marital status	Married	0.165 **		Friends	0.102 **
	Separated	−0.066 **	Network contact	Children	0.148 **
	Divorced	−0.137 **		Relatives	0.101 **
	Widowed	−0.031 **		Friends	0.116 **
	Single	−0.095 **	Mood	Optimism	0.390 **
Subjective social status		0.406 **		Pessimistic	−0.337 **
Social activities		0.218 **		Positive emotion	0.478 **
Personality trait	Dutifulness	0.222 **		Negative emotion	−0.439 **
	Openness	0.284 **		Hopelessness	−0.405 **
	Extroversion	0.284 **	Living conditions	Neighborhood relations	0.287 **
	Agreeableness	0.155 **		Environmental identity	0.208 **
	Neuroticism	−0.325 **			

** *p* < 0.01.

**Table 4:** Feature selection of predictors related to life satisfaction.

*α*	0.1	0.05	0.03	0.01	0.001
Training-set fitting index (*R*^2^)	0.419	0.420	0.421	0.422	0.422
Test-set fitting index (*R*^2^)	0.446	0.447	0.448	0.448	0.448
Number of predictors	14	16	17	18	18

*α* is the coefficient of some non-significant predictors, *R*^2^ is the index for the model fit.

**Table 5:** Results of model fitting with life satisfaction as the dependent variable.

Dataset	Indictor	Linear Model			Nonlinear Model		
		MLR	RR	LASSO	SVR	KNN	DT
Training set	*MAE*	4.392	4.392	4.392	4.155	4.054	4.324
	*MSE*	32.796	32.796	32.796	32.173	28.452	31.815
	*R* ^2^	0.430	0.430	0.430	0.441	0.506	0.447
Test set	*MAE*	4.339	4.339	4.339	4.151	4.488	4.595
	*MSE*	32.579	32.579	32.579	32.416	34.961	36.444
	*R* ^2^	0.433	0.433	0.433	0.436	0.392	0.366

**Table 6:** Linear regression coefficients of predictors.

Predictors	Dimension	Coefficient (*β*)	Predictors	Dimension	Coefficient (*β*)
Subjective social status		1.522 **	Mood	Optimism	1.067 **
Social activities		0.416 **		Pessimistic	0.168 **
Positive support	Spouse	0.594 **		Positive emotion	1.227 **
	Children	0.376 **		Negative emotion	−1.185 **
Negative support	Spouse	−0.826 **		Hopelessness	−0.478 **
	Children	−0.276 **	Personality trait	Neuroticism	0.268 **
Intimacy	Spouse	0.686 **		Extroversion	0.294 **
Living conditions	Neighborhood relations	0.482 **		Openness	−0.779 **
	Environmental identity	0.042		Dutifulness	−0.040
			Intercept		24.878 **

** *p* < 0.01.

**Table 7:** Important feature of life satisfaction in decision-tree regression model.

Predictors	Dimension	Coefficient (*β*)	Predictors	Dimension	Coefficient (*β*)
Subjective social status		0.092	Mood	Optimism	0.056
Social activities		0.004		Pessimistic	0.001
Positive support	Spouse	0.138		Positive emotion	0.407
	Children	0.012		Negative emotion	0.114
Negative support	Spouse	0.078		Hopelessness	0.042
	Children	0.006	Personality trait	Neuroticism	0.000
Intimacy	Spouse	0.015		Extroversion	0.000
Living conditions	Neighborhood relations	0.013		Openness	0.008
	Environmental identity	0.003		Dutifulness	0.001

## Data Availability

The data presented in this study are public and available to researchers. The dataset is distributable only by RAND Health and Retirement Study (RAND HRS) team and available in public registrations on the RAND HRS website: https://hrsdata.isr.umich.edu/data-products/rand (accessed on 30 December 2020), and is also available upon reasonable request from the corresponding authors.
